# ESBL-Producing Strain of Hypervirulent *Klebsiella pneumoniae* K2, France

**DOI:** 10.3201/eid2209.160681

**Published:** 2016-09

**Authors:** Laure Surgers, Anders Boyd, Pierre-Marie Girard, Guillaume Arlet, Dominique Decré

**Affiliations:** Assistance Publique Hôpitaux de Paris, Hôpital Saint-Antoine, Paris, France (L. Surgers, P-M. Girard, G. Arlet, D. Decré);; Université Pierre et Marie Curie, Sorbonne Universités, Paris (L. Surgers, A. Boyd, P-M. Girard, G. Arlet, D. Decré)

**Keywords:** Klebsiella pneumoniae, Enterobacteriaceae, hypervirulent, K2, ESBL, CTX-M-3, bacterial infection, bacteria, France

**To the Editor:** *Klebsiella pneumoniae* is mainly responsible for hospital-acquired urinary tract infections, bacteremia, pneumonia and intra-abdominal infections. However, since the mid-1980s, *K. pneumoniae* has also been described as the cause of highly invasive community-acquired infections (*1**,**2*). The *K. pneumoniae* isolates associated with such infections are often hypermucoviscous and frequently belong to the capsular serotypes K1 or K2. Two of the most extensively studied genes associated with invasive infections are a mucoviscosity-associated gene A (*mag*A) in serotype K1 and a regulator of mucoid phenotype A (*rmp*A). These strains of hypervirulent *K. pneumoniae* (hvKP) are now circulating worldwide (*1*,*2*).

At the same time, a substantial increase of high-level antimicrobial resistance acquired by non-hvKP strains has also been observed. Clonal complexes of hvKP and multidrug-resistant (MDR) strains had been considered independent (*3*) until 2014, when extended-spectrum β-lactamase (ESBL)– or carbapenemase-producing hvKP were first identified in China (*4*). Here we report an ESBL-producing strain of hvKP isolated from a patient in France.

The patient was a 56 year-old woman, born in Algeria, who alternately resided in France and Algeria for several years without travel to any other country. She underwent liver transplant in 2007 for primary biliary cirrhosis. In 2012, she had a routine posttransplant liver biopsy indicating granulomatosis, resulting in several examinations to determine its etiology. The patient was afebrile with no inflammatory syndrome. A thoracic computed tomography scan indicated no abnormalities. We performed a bronchoalveolar lavage, and bacteriologic examination of the specimen indicated a strain of *K. pneumoniae* with a hypermucoviscosity phenotype. We identified the ESBL phenotype with a positive double-disk synergy test between clavulanic acid and third-generation cephalosporins and aztreonam. We conducted antimicrobial susceptibility tests according to guidelines issued in 2013 by the Comité de l’Antibiogramme de la Société Française de Microbiologie (http://www.sfm-microbiologie.org/UserFiles/files/casfm/CASFM2013vjuin.pdf). These tests revealed high-level resistance to third-generation cephalosporins and to all aminoglycosides except amikacin. The strain was susceptible to cefoxitin, piperacillin/tazobactam, carbapenems, all fluoroquinolones, fosfomycin, and trimethoprim/sulfamethoxazole. The patient did not receive any antimicrobial drug treatment and after 3 years of follow-up reported no signs of pneumopathy.

Multiplex PCR and sequencing of the identified strain indicated the presence of *bla*_CTX-M-3_ (*5*). We extracted plasmid carrying the ESBL gene with the DNA Plasmid Miniprep Kit (QIAGEN, Valencia, CA, USA) and transferred the gene by electroporation into *Escherichia coli* DH10B cells. Relaxase typing, which detects major replicon groups, revealed only IncL/M plasmid (*6*). We performed another multiplex PCR to determine capsular serotypes K1 or K2 and the presence of major virulence factors. The capsular serotype was K2 (*7*). We identified *rmp*A, the plasmid-mediated gene regulating extracellular polysaccharide synthesis, and *iut*A, *ent*B, *mrk*D, and *ybt*S genes by this PCR test. The isolate belonged to sequence type (ST) 86 as determined by multilocus sequence typing (http://www.pasteur.fr/mlst).

The earliest-described strains of hvKP, which were isolated from liver abscesses, were predominantly serotype K1 and ST23 (*2*). The most frequently isolated non-K1 hvKP stains are currently serotype K2, known to cause hepatic abscesses and severe cases of pneumonia and other hvKP-associated infections such as necrotizing fasciitis (*2*). The K2 strains isolated to date appear to originate from a much broader range of ST groups that include ST86 and many others (e.g., ST65, ST66, ST373, ST374, ST375, ST380, and ST434) (*8*,*9*).

This patient, despite being immunocompromised, was only colonized with what is normally considered a highly pathogenic strain. In general, the acquisition of antimicrobial resistance genes reduces fitness, which could have been the case in this patient. However, other strains carrying MDR and high-virulence genes, namely *E. coli* ST131, have no loss in fitness (*10*). These clones might harbor other biologic factors providing a competitive advantage.

MDR strains of *K. pneumoniae* have emerged in recent years and have been identified as a major threat to public health by the US Centers for Disease Control and Prevention. hvKP, with its high pathogenic potential, has also been on the rise during the same period. In the past, MDR and hvKP strains evolved separately in distinct clonal groups (*2*), but the recent emergence of hvKP harboring the gene for MDR, such as the one identified in our study, raises newfound concerns. Our patient was colonized with an ST86 CTX-M-3–producing strain of K2 hvKP, raising the question of whether MDR hvKP strains could circulate in Europe.
